# Book Review

**Published:** 1945

**Authors:** 


### Reviews of Books

**The Medical Annual, 1945.** Edited by Sir Henry Tidy, K.B.E., and A. Rendle Short, M.D., F.R.C.S. Pp. lxxviii., 410. With 47 plates. Bristol: John Wright & Sons Ltd. Price 25s.—This admirable conspectus of the progress of medicine is the sixty-third volume of the Series. Two wars have not interrupted its yearly publication, and we are glad to see that the Ministry of Supply has allotted to the publishers sufficient paper to permit of a slight increase in the size of the issue. There is one subject which although not so very new is included for the first time in the *Medical Annual*, namely "Vital Statistics." This is reviewed by Dr. Percy Stocks, Medical Statistician at the General Register Office, Somerset House, who was for a time a School Medical Officer in Bristol. His review deals with four broad groups of figures dealing with Population, Natality, Mortality and Morbidity: and the facts he sets out are not nearly so dull as might be anticipated. He makes figures talk! In Therapeutics one of the most recent advances discussed is the value of penicillin in Gonorrhœa and Syphilis. There is also an excellent general review of penicillin. The astonishing success of ligation of the patent ductus arteriosus in children is briefly but clearly described. The article on Diabetes Mellitus seems somewhat disinclined to admit the value of globin insulin, possibly longer experience will tend to bring this preparation into greater favour. A thoughtful evaluation of the Kenny treatment of Poliomyelitis is contributed by Macdonald Critchley. One of the longest and most instructive articles is that on Primary Atypical Pneumonia by Colonel William S. Middleton of the U.S. Army Medical Corps. The pros and cons of Mass Miniature Radiography of the Chest are set out in considerable detail and with laudable impartiality. Wright's *Medical Annual* must be congratulated on having come successfully through exceptional trials during the war and we hope that within a year or two it may be restored to its former size, thus extending its acknowledged usefulness.

**Notable Names in Medicine and Surgery.** By Hamilton Bailey and W. J. Bishop. Pp. viii., 202. Price 15s.—This is a most interesting little book. It gives a very brief sketch of the careers of about eighty famous physicians and surgeons, ranging from Hippocrates and Galen to Gallie and Böhler. Needless to say, the treatment is very sketchy, two or three pages being given to each. There is usually a photograph and often other illustrations besides. The descriptions are lighthearted and chatty. Surely no where else can one find so much information about so many people in so small a compass.

**The Life and Teaching of Sir William MacEwen.** By A. K. Bowman, M.B., Ch.B. Pp. xii., 425. Edinburgh: William Hodge & Co. 1942.

Price 21s.—This is a most appreciative account of the life and work of MacEwen, written by Dr. Bowman, Regional Medical Officer of Health for Scotland, who was at one time one of MacEwen's pupils. There are very few men of our day of whom it could truly be said, as of MacEwen, that a history of his life was a chapter of the history of modern surgery. In this respect he compares with the Mayos and Moynihan. In almost every branch of surgery he was a pioneer and a leader. A pupil of Lister, and following him as he did in the Chair of Surgery of Glasgow, it was natural that he should be one of the leaders of the new doctrine of antiseptics. He was an orthopedic surgeon, long before that term was used and his work in transplantation of bone and the growth of bone was of an epoch-making and enduring character, whilst its scientific value was at once recognized and led to his being elected as a Fellow of the Royal Society. In general surgery he did bold work on the subject of the treatment of aneurysm, he was one of the first to treat laryngeal stenosis by intubation. But there are two special branches of surgery in which he became leader and teacher; these were cerebro-spinal and lung surgery. In the former it was he who did so much pioneer work about the origin of septic disease of the brain and meninges. In the latter his success in partial or total extirpation of the lung leaves us in a state of wonder and amazement that such heroic measures should have been carried out without the aid of X-rays, or the help of modern instruments.

**Elementary Morphology and Physiology.** Third Edition. By J. H. WOODGER, D.Sc. Pp. xvi., 522. Illustrated. London: Oxford University Press. 1943. Price 15s.—This is a textbook of zoology suitable for the student reading for the first M.B. It is not a dissecting manual, and presupposes the use of such by the dissector. It gives a description of a considerable number of animal types of interest to the medical student, and discusses in a simple manner the theoretical relations between them and the problems of function. Some chapters, such as that on genetics, are not so simple. There is a brief reference to the development in past geological time of some of the animals discussed. The illustrations are mostly of the nature of diagrams of microscopic sections. Embryology is carefully considered. To doctors who have a taste for biology this book can be commended, as well as to students.

**Tropical and Sub-tropical Diseases.** By C. H. BARBER, D.S.O., M.D. Pp. x., 189. Illustrated. London: Oxford University Press. 1942. Price 5s.—This little book of 189 pages is intended to be used for reference by medical men serving with the Forces in the tropics. In so small a compass one cannot expect a full account of any disease, and one would expect the author to confine himself to some entirely practical instructions for diagnosing and treating the commoner disorders likely to occur among the troops. It is disappointing, therefore, to find that an attempt has been made to say a few words about a very large number of conditions, some rare and others unimportant. More than a page is devoted quite unnecessarily to the history of malaria and a few lines, often inaccurate and out of date, are given to the pathology of each disease mentioned. The instructions for treatment are equally brief and while generally omitting to mention the strength of solutions recommended often include tit-bits of historical interest. We hope that no one will adopt the practice, recommended by the author, of burning the houses of natives suffering from yaws : the measure is unnecessary and likely to lead to unfortunate political developments. In short, the book is without merit and in our opinion was not worth publishing.

**Penicillin in Warfare.** *Special Issue of The British Journal of Surgery, Vol.* XXXII. Pp. 112. Illustrated. Bristol : John Wright & Sons Ltd. 1945. Price 12s. 6d.—This issue of 112 pages is a mine of information on the part played by Penicillin in traumatic surgery. It consists of a series of articles by surgeons and pathologists, acknowledged experts in their special fields, who have had experience of the use of Penicillin in all manner of war injuries involving soft tissues, bones, the thorax, the head and the spine. There are many illustrations and the text is full of detailed directions for the proper treatment of conditions that occur not only in warfare, but also in civilian practice. The treatment of gas gangrene is described and there are notes on the use of Penicillin in gonorrhœa and syphilis. A valuable feature of most of these articles is the attention paid to the necessary bacteriological control of treatment, and the reader may well be surprised to see what care was taken to study the bacterial flora throughout all stages of the cases here described. In one of the first papers Lieut.-Col. Jeffrey draws attention to the limitations and misuses of penicillin and the same scrupulous standards are maintained by all the authors of this most valuable symposium. The appearance of this special number of the *British Journal of Surgery* is most timely ; it provides the busy surgeon with an admirably handy guide to the role of Penicillin in traumatic surgery.

**Physical Signs in Clinical Surgery.** Ninth Edition. By HAMILTON BAILEY, F.R.C.S. Pp. viii., 351. Illustrated. Bristol : John Wright & Sons Ltd. 1944. Price 25s.—This book is chiefly remarkable for the wonderful collection of illustrations, many of them in colour. This commendation must not be taken as any disparagement of the text, which is also excellent. It would be difficult to think of another book which could be as useful to student, general practitioner, or surgeon, in enabling him to make a diagnosis from clinical signs, both of the more common and less common surgical diseases and accidents. A pleasing feature is the line of description in the footnotes of each surgeon or pathologist whose name is mentioned.

**Pye's Surgical Handicraft.** Fourteenth Edition. By HAMILTON BAILEY, F.R.C.S. Pp. xi., 628. Illustrated. Bristol : John Wright & Sons Ltd. 1944. Price 25s.—This book is described as a manual of minor surgery and matters connected with the work of surgical dressers, house surgeons and practitioners. It is an invaluable book to this section of the profession, and is in effect a comprehensive collection of the details of a great number of technical procedures. If anything, it is too comprehensive and in consequence some parts are less full than others. It is an excellent work which quite definitely supplies a need amongst the section of the profession to which it is addressed. The illustrations are numerous and excellent, and the book is well set out. The type and paper are up to pre-war standards, but it is to be regretted that few if any references are given.

**Diseases of the Eye.** Second Edition. By EUGENE WOLFF, M.B., B.S., F.R.C.S. Pp. xii., 222. Illustrated. London: Cassell & Co. Ltd. 1942. Price 21s.—In the second edition of his text-book for students and practitioners, the author, while retaining all the features which made its predecessor so acceptable as a guide to the beginner in ophthalmology, has now added to them by the inclusion of additional illustrations, further details of treatment, and a chapter on bandaging. He is well aware of the practical difficulties in teaching the subject, and has been most successful in presenting it in an interesting form. The text includes descriptions of all the usual (and some of the less common) eye diseases, of methods of examination, of correcting errors of refraction, and of the standard major and minor ophthalmic operations. Brief but adequate notes on anatomy and pathology are augmented by excellent annotated drawings and reproductions of microscope sections. The illustrations, comprising 128 in the text and five colour plates, are particularly good, while the large size of page allows of their convenient placing in reference to the descriptive matter. This is a very attractive volume, and can be recommended with confidence to those for whom it was written as one of the best shorter ophthalmic text-books.

**Industrial Toxicology.** By DONALD HUNTER, M.D., F.R.C.P. Pp. 80. London: Oxford University Press (Humphrey Milford). Price 10s. Dr. Hunter's Croonian Lectures for 1942, which have already been published in the *Quarterly Journal of Medicine*, now appear in very handsome book form. These lectures give a detailed review with full bibliography of the effects of toxic substances encountered in industry. The information is of the greatest importance to industrial medical officers as well as to certifying surgeons and medical referees under the Workmen's Compensation Acts. Every practitioner will be glad to know where to turn to in cases of doubtful industrial diseases from which his patients may be or may claim to be suffering. This is a work of great authority.

**Introduction to Diseases of the Chest.** Second Edition. By JAMES MAXWELL, M.D., F.R.C.P. Pp. xii., 292. Illustrated. London: Hodder & Stoughton Ltd. 1945. Price 12s. 6d. net. The principal change in this second edition of a book that is an established favourite consists in presenting the X-ray illustrations as negatives. This is the form in which they are studied in actual practice and is a great improvement. We could wish that Dr. Maxwell (with Gee's little book beside him) had re-written his Chapter on auscultation and had not persisted in spelling sibilus with a double "l."

**Pharmacology.** Second Edition. By J. H. GADDUM, D.Sc., M.R.C.S., L.R.C.P. Pp. xvi., 436. Illustrated. London: Oxford University Press. 1944. Price 21s.—In the second edition of this book, Professor Gaddum shows himself to be a very reliable guide among the intricacies of modern pharmacology. Clearly, concisely, and lighting the way with flashes of humour, he leads us through the experimental basis of pharmacology, warns us of the snags, statistical and others, displays much useful information in charts, tables, and structural formulae, and leaves us with a "key to the interpretation of chemical names," so that in the future we can venture into the promised land of therapeutics. On his way he deals with almost every substance of real use in medicine and makes kindly mention of many old and favourite drugs not always so favoured these days. The peripheral neuritis of lead and the escape of the brachioradialis is an old skeleton that he might well cover up, and poor Thomas Dover is libelled once again, the toxicity of Lewisite is under estimated, and so is the ease with which mercurial diuretics will clear the body of Vitamin B, but these are small points to cavil at in a book that will be widely welcomed. Teachers will welcome it as the only small book that will give students an insight into the experimental side of pharmacology, and the students will value it for its lucidity and ease of reading, and practitioners should welcome it as a handy reference to the best of both old and new remedies.

**Treatment of Shock.** By R. W. RAVEN, F.R.C.S. Pp. 96. Illustrated. London: Oxford University Press. 1942. Price 5s.—Those who remember the Oxford War Primers of 1914-18 will find this present series of War Manuals, to which this book belongs, maintaining the same high standard. In this small book shock is very adequately described. All the latest advances receive attention. The pathology and treatment of secondary shock are fully given, whilst methods of oxygen administration, blood and plasma transfusion are described. Anæsthesia and surgical shock also receive attention. The book is of handy pocket size, well illustrated and printed. The list of references at the end should prove most useful. Anyone desiring a record of this subject in concise and practical form will find this book invaluable.

**Tuberculosis in Childhood.** First Edition. BY DOROTHY STOPFORD PRICE, M.D. Pp. vii., 215. Illustrated. Bristol: John Wright & Sons Ltd. 1942. Price 17s. 6d. net.—This unpretentious looking volume is packed full of information. The author has a very extensive background of experience, and has covered clearly and adequately the ground indicated by the title. It is a very valuable book for anyone looking for a comprehensive and authoritative account of the subject. Ranke's classification into three stages is adopted and justified by an imposing weight of illustration and argument. The tuberculin skin reaction is made the main foundation in diagnosis. The facts about tuberculin surveys are impartially set out; some of the deductions are not self-evident. Incidentally, it is a curious reminder of the neutrality of Eire to find the Hamburg ointment "produced by Dr. Fresenius, Frankfort-on-Main, Germany," recommended as the one to use in diagnosis. The volume concludes with an admirable chapter by Mr. H. F. Macauley on tuberculous lesions of bones and joints, which in less than thirty pages gives a more intelligible account of the subject than some books devoted to it. There is a very useful bibliography and index. This is a book which those interested in tuberculosis cannot afford to overlook.

**A Guide to the Surgical Paper with Questions and Answers.** Second Edition. By R. J. MCNEILL LOVE, M.S., F.R.C.S. Pp. 79. Illustrated. London: H. K. Lewis & Co. Ltd. 1944. Price 6s.—This book is intended as a help to a candidate faced with a written paper in a surgical examination. In his introduction, the author gives a few useful tips which may seem obvious, but are, nevertheless, frequently neglected, such as allowing oneself enough time for each question and trying to grasp the meaning of the question. He then proceeds to discuss various terms recurring time and again in examination papers. The bulk of the book consists of thirty specimen questions to which skeleton answers are supplied in a sealed part of the book. These answers are necessarily very condensed and schematic. This book is certainly most useful as a short guide to any candidate for a written surgical examination and should help candidates to do themselves justice in their surgical papers.

